# Study of Conduction Blocks in ST Elevation Myocardial Infarction – A Cross-Sectional Analysis

**DOI:** 10.5152/eurasianjmed.2024.20164

**Published:** 2024-10-01

**Authors:** Swapnil Shinde, Nitin Jadhav

**Affiliations:** Department of General Medicine, Krishna Institute of Medical Sciences University, Maharashtra, India

**Keywords:** Atrioventricular block, coronary artery disease, diabetes, heart block, hyperlipidemia, hypertension

## Abstract

**Background:**

Conduction blocks complicating ST(ST-segment)-elevation myocardial infarction are associated with increased morbidity and mortality. Research indicates that anterior and inferior wall myocardial infarction were the most encountered causes of blocks but with conflicting results. However, patterns of conduction blocks have not been widely established in our population. The aim was to study the various patterns of conduction blocks following ST-elevation myocardial infarction and their prognostic implications.

**Methods:**

Prospectively, 70 patients, aged > 18 years, diagnosed with ST segment elevation myocardial infarction were included in the study. Post intensive care unit admission, all patients were observed for conduction blocks using a standard 12-lead electrocardiogram and repeated the same every 48 h throughout the hospitalization stay. Statistical analysis was performed using software R version 3.6.0.

**Results:**

Out of 70 patients, 70% were males. Mean age was 60.7 ± 13.4 years. The proportion of blocks was first-degree heart block (28.6%), Mobitz II heart block (20%), complete heart block (17.1%), Mobitz I heart block (11.4%), right bundle branch block (10%), left bundle branch block (10%), left anterior hemiblock (1.4%), and trifascicular block (1.4%). No significant difference was found between males and females with respect to various conduction heart blocks (*P *> .05). Mortality was observed only in patients with complete heart block (11.4%) and first-degree heart block (2.8%; *P *= .003). Statistically, no significant difference was observed between various conduction blocks with respect to cardiac enzymes, random blood sugar, and lipid levels (*P *> .05).

**Conclusions:**

High mortality rate has been found in the patients with complete heart block indicating that severity of conduction block is a predictor of poor outcome in the ST-elevation myocardial infarction patients.

**Main Points**

- Acute myocardial infarction (AMI) is one of the major problem.
  Conduction blocks are frequent complications of AMI.
- Bundle branch block in AMI carries poor prognosis.This has been attributed both to the extent of myocardial damage and to the frequency of ventricular asystole.
- Development of conduction blocks worsens the outcome of AMI. Knowledge about various types of conduction blocks occurring in AMI helps in early recognition of conduction blocks, so that appropriate treatment including pacing can be instituted at an early stage.

## Introduction

ST-segment elevation myocardial infarction (STEMI) is a grave medical condition and remains to be a leading cause of mortality globally.^1^ The STEMI is due to blockage of one or more coronary arteries. This acute blockage of blood flow to the heart is due to the factors like plaque rupture, erosion, fissuring, or dissection of coronary arteries which forms as obstructing thrombus. The major causes due to this condition are dyslipidemia, diabetes mellitus, hypertension, smoking, and family history of coronary artery disease.^2^

STEMI can lead to different complications, such as conduction blocks, ventricular dysfunction, cardiogenic shock, mechanical complications, and ventricular arrhythmias.^2^ However, the prognosis of STEMI patients developing these complications is very poor. Cardiac conduction blocks are the electrical disturbances which may occur following acute myocardial infarction (MI). Delayed or interrupted conduction may occur as a result of physiological changes; ischemia causing temporary or permanent structural changes of the tissues surrounding the sinoatrial (SA) node and atrioventricular (AV) junctions, increased parasympathetic tone commonly associated with an inferior wall MI, increased extracellular potassium that slows down the cardiac impulse conduction, and local release and formation of that decelerates the impulse conduction through AV node.^3^

Development of heart blocks may worsen the outcome of STEMI. Knowledge regarding different types of conduction blocks occurring in STEMI may help in the early recognition of patients at increased mortality risk, so that appropriate treatment including temporary or permanent pacing can be instituted at an early stage.^4^ Mortality rate in our country is considerably lower than developed countries which may be due to higher younger population rate than those in developed countries.^5-7^ However, the studies on the pattern of conduction blocks have not been widely established in our population. Therefore, this study was intended to study the pattern of conduction blocks following STEMI and its prognostic implications at tertiary care hospital. So, this study findings would help physicians in practice for the management of STEMI patients at high risk in order to reduce the morbidity and mortality.

## Material and Methods

After obtaining ethical clearance from KIMSDU, Karad (KIMSDU/IEC/03/2017; Protocol no: 011/2017-2018 Dated: November 23, 2017), this prospective, observational study was carried out for 2 years between December 2017 and May 2019 in the Department of General Medicine and Intensive care unit (ICU) at a tertiary care teaching hospital, Karad, Maharashtra, India. Informed consent was obtained from the participants who agreed to take part in the study.

By non-probability (consecutive) sampling technique, a total of 70 patients aged > 18 years diagnosed with STEMI as per World Health Organization criteria (clinical history of typical chest pain lasting >30 minutes, classical echocardiogram (ECG) changes consistent with acute MI, and elevated cardiac enzymes levels of creatine kinase (CK-MB) and troponin I) were included after attaining written informed consent form. Patients with old bundle branch block, cardiomyopathy or established valvular heart disease, congenital or rheumatic heart disease, and medication history of drugs (viz. clonidine, methyldopa, verapamil, and digoxin) inducing conduction blocks were exempted from the study.

Data was collected for gender-Wise Distribution of Symptoms and Risk Factors in STEMI Patients as per table number I which included detailed history of chest pain, risk factors and their duration, Medication. Random venous blood sample was obtained for the analysis of cardiac enzymes (CK-MB [EM360 analyzer] and troponin I [cTnI; EM360 analyzer]), blood glucose, and lipid profile. A diagnosis of STEMI was confirmed by chest pain lasting>30 minutes, ST-segment elevation ≥1 mm in at least 2 of the limb leads, and elevation of CK and its myocardial band (MB) fraction to more than twice the upper limit of normal or troponins. Upon admission into ICU, a standard 12-lead ECG (at a paper speed of 25 mm/s and an amplification of 10 mm/mV) was recorded and repeated the same every 48 hours throughout the hospitalization stay.

### ECG Evaluation

New ST elevation at J-point in 2 contiguous leads with cut points: ≥0.1 mV in all leads other than leads V2-V3 (≥0.2 mV in men ≥ 40 years, ≥0.25 mV in men < 40 years, and ≥0.15 mV in women). The conduction blocks based on ECG characteristics could be categorized as follows: atrioventricular block (including first-degree AV block, Mobitz type I, Mobitz type II, and third-degree/complete AV block), and intraventricular blocks (including left bundle branch block, right bundle branch block [RBBB], left anterior hemiblock, and left posterior hemiblock).

Data regarding CK-MB, cTnI, random blood glucose (RBS), lipid profile (total cholesterol [TC] and triglycerides [TG]), and infarction site were recorded. A follow-up was done for all patients till hospital discharge.

### Statistical Analysis

Statistical analysis was done using software R version 3.6.0. Normality of the data was determined using the Shapiro–Wilk test. The continuous variables with normal distribution were presented as mean ± standard deviation and compared using paired *t*-test. Kruskal–Wallis test was performed for variables without normal distribution. The categorical variables were presented as frequencies and percentages and compared using proportion z-test. A *P* value of <0.05 was considered statistically significant at 95% CI.

## Results

Out of 70 patients with STEMI, males (70%) constitute significantly higher proportion compared to females (30%; *P* < .00001). The mean age was 60.7 ± 13.4 years. The majority (64.3%) were in the age group 51-70 years. Table 1 depicts the gender-wise distribution of symptoms and risk factors among study participants. Table 2 shows the frequency of different infarction sites and conduction blocks in our study population. Statistically, no significant difference was found between males and females with respect to various conduction heart blocks (*P* > .05; Graph 1). Though there was no significant difference, a higher number of patients among all conduction blocks were of male gender. Mortality was observed only in patients with complete heart block (11.4%) and first-degree heart block (2.8%); significantly higher in complete heart block than first-degree block (*P *= .003), while a marked improvement was noted in those with other conduction blocks. The period of hospitalization of all study participants ranged from 4 to 12 days with a mean of 6.7 days. Statistically, no significant difference was observed between various conduction blocks with respect to laboratory investigations, such as cardiac enzymes (CK-MB and cTnI), RBS, and lipid profile (*P* > .05; Table 3). days. Table 4 depicts the mortality pattern according to Killip classification.

## Discussion

STEMI can lead to different complications, such as conduction blocks, cardiogenic shock, and ventricular arrhythmias. Conduction blocks complicating STEMI are associated with increased morbidity and mortality.^1,2^ This study has analyzed the various conduction blocks associated with STEMI in 70 patients and the majority were in the 51-70 years age group, with a male predominance (70%). This is comparable to Ratan Ram et al^8^ where 51-60 years age group were most affected with STEMI, indicating that middle-aged males were more prone to STEMI. Vijay Kumar et al^9^ reported mean age of patients with conduction blocks was 62.9 years, whereas in patients without blocks, it was 57.4 years. On comparison with previous studies (especially non-Asian studies), our study patients were younger, corroborating with evidence from recent studies, that CAD occurs 10 years earlier in the Indian population.^10,11^

Here, hypertension (38.6%) was the most common risk factor found in STEMI patients, followed by diabetes (27.1%). Ratan Ram et al^8^ reported that hypertension (27%), diabetes (25%), and smoking (30%) were the most encountered risk factors, whereas Chavda et al^12^ reported smoking (72.0%), followed by IHD (14%) and diabetes (10%).

The most prevalent infarction site was the anterior wall (38.6%), followed by inferior wall (34.3%). In contrast, Ratan Ram et al^8^ reported that inferior wall was the most common site of MI, followed by anterior wall MI. However, our study findings were consistent with Hreybe and Saba and Shah et al.^13,14^

Eight different types of conduction blocks have been observed with first-degree heart block being the most prevalent one equally in anterior and inferior wall MI, followed by Mobitz type II AV heart block and complete heart block. Similarly, Ratan Ram et al^8^ reported higher prevalence of first-degree AV block (7%), followed by second-degree AV block (4%) and complete heart block (3%). On the contrary, previous studies reported a significantly higher incidence of first-degree heart blocks with inferior wall MI than anterior wall MI.^13^ In the present study, gender-wise distribution of conduction blocks was studied and no significant difference was found. Though there was no significant difference, the higher number of patients among all conduction blocks was of male gender. Ratan Ram et al^8^ observed that males had more conduction blocks than females (18.0% vs. 14.7%) as observed in this study. 

High mortality rate was observed in patients with complete heart block (11.4%), followed by first-degree heart block (2.8%). Patients with complete heart block are at a higher risk of developing ventricular tachycardia, asystole, and sudden cardiac death, while a marked improvement was noted in those with other conduction blocks. Ratan Ram et al^8^ reported a mortality rate of 41.2% (7/14) and 16.8% (14/83) among STEMI patients with and without conduction blocks, respectively; indicating that acute MI patients with conduction block had higher chance of mortality compared to non-conduction block MI patients. Similarly, Vijay Kumar et al^9^ also reported higher mortality in STEMI patients with blocks (19.1%) compared to STEMI without blocks (2.5%). A higher incidence of conduction blocks was found in patients with anterior wall MI (38.6%), followed by inferior wall MI (32.8%). In contrast to our study findings, Majumdar AA et al^15^ and Vijay Kumar et al^9^ reported a significantly higher incidence of conduction blocks with inferior wall MI (66.7%) compared to anterior wall MI (66.7% vs 33.3% and 56.8% vs 31.8%; *P* < .05, respectively). Out of 4 patients with RBBB, 2 patients expired with a mortality of 50%, which is in concordance with 52% as in Godman et al.^16^ The mortality was higher in males (60%) compared to females (40%). Similar findings were reported by several studies.^17^ Hence, male patients with complete heart blocks had higher probability of mortality compared to female patients. No significant difference was observed between various conduction blocks with respect to laboratory investigations, such as cardiac enzymes (CK-MB and cTnI), RBS, and lipid profile (TC and TG). None of the studies related these investigations with respect to various conduction blocks.

Moreover, the mortality rate in our study population was considerably lower than studies conducted in developed countries which may be due to higher younger population rate than those in developed countries.^5-7^ On the other side, the overall prevalence of conduction blocks is more common in our population compared with developed countries.^18^ Hence, more precise studies with all underlying factors contributing to this need should be encouraged to confirm this hypothesis. Our study had its own limitations in that we did not consider non-conduction block patients for comparison as we directly recruited STEMI patients with recent conduction blocks. Second, we did not follow up the patients after hospital discharge.

High mortality rate has been found in the patients with complete heart block indicating that severity of conduction block is a predictor of poor outcome in STEMI patients. All patients with STEMI should be monitored for early recognition of conduction blocks and appropriate treatment should be started to improve the outcome of patient. In addition, future studies should focus on non-conduction block patients for comparison with regular follow-up.

## Data Availability Statement

The data that support the findings of this study are available on request from the corresponding author.

## Figures and Tables

**Figure 1.**
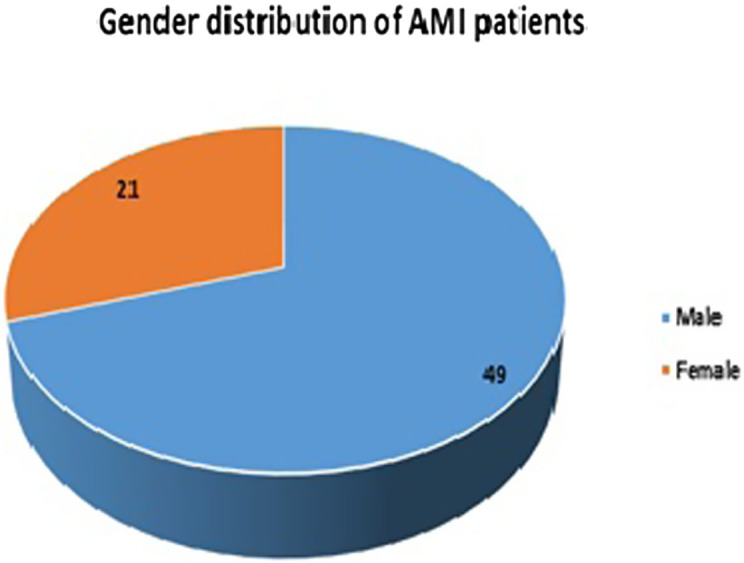
Gender-wise distribution of conduction blocks in STEMI patients.

**Table 1.** Gender-Wise Distribution of Symptoms and Risk Factors in STEMI Patients

Variables		Gender, n (%)		Frequency (%)	P*
		Male	Female		
Symptoms	Chest pain	49 (100)	20 (95.2)	69 (98.6)	.661
	Vomiting	20 (40.8)	6 (28.5)	26 (37.1)	.482
	Sweating	48 (98)	19 (90.5)	67 (95.7)	.439
	Dyspnea	25 (51)	10 (47.6)	35 (50)	1
	Palpitation	11 (22.4)	8 (38.1)	19 (27.1)	.291
Total		49	21	70	-
Risk factors	Hypertension	20 (40.8)	5 (23.8)	25 (35.7)	.276
	Diabetes	13 (26.5)	6 (28.6)	19 (27.1)	1
	Alcohol	15 (30.6)	0	15 (21.4)	.005
	Cerebral vascular accident	0	1 (4.8)	1 (1.4)	.66
Total		49	21	70	-

*Proportion z-test.

**Table 2.** Distribution of Patients According to Site of Infarction and Conduction Block Type

Site of Infarction	Frequency (%)	Percentage
Anterior wall MI	27 (38.6)	38.6
Inferior wall MI	24 (34.3)	34.3
Lateral wall MI	8 (11.4)	11.4
Anterolateral wall MI	5 (7.1)	7.1
Anteroseptal wall MI	3 (4.3)	4.3
Inferoposterior wall MI	2 (2.9)	2.9
Anteroinferior wall MI	1 (1.4)	1.4
Block type	**Frequency (%)**	**Percentage**
First-degree heart block	20 (28.6)	28.6
Mobitz type II heart block	14 (20)	20
Complete heart block	12 (17.1)	17.1
Mobitz type I heart block	8 (11.4)	11.4
Right bundle branch block	7 (10)	10
Left bundle branch block	7 (10)	10
Left anterior hemi block	1 (1.4)	1.4

MI, myocardial infarction

**Table 3.** Distribution of Conduction Blocks Based on Site of Infarction

Infarction site	Blocks (n)	CHB	First Degree HB	LAHB	LBBB	MT 1 HB	MT 2 HB	RBBB	TB
Anterior Wall MI	27	3	9	0	1	1	7	6	0
Anteroinferior wall MI	1	1	0	0	0	0	0	0	0
Anterolateral MI	5	0	0	0	1	0	3	1	0
Anteroseptal wall MI	3	1	0	0	0	1	1	0	0
Inferoposterior wall MI	2	1	0	0	0	1	0	0	0
Inferior wall MI	23	5	9	0	2	5	2	0	0
Lateral wall MI	6	1	0	0	3	0	1	0	1

CHB, complete heart block; HB, heart block; LAHB, left anterior hemi block; LBBB, left bundle branch block; MI, myocardial infarction; MT 1 HB, Mobitz type 1 heart block; MT 2 HB, Mobitz type 2 heart block; RBBB, right bundle branch block; TB, trifascicular block.

**Table 4.** Mortality Pattern Based on Killip Classification and Gender

Killip Class	Frequency (n = 70 )	Mortality		Gender	
		Frequency	Percentage	Male (n)	Female (n)
Class I	44	1	2.3	1	0
Class II	19	2	10.5	0	2
Class III	6	6	100	4	2
Class IV	1	1	100	1	0

**Table 5.** Comparison of Cardiac Enzymes and Lipid Levels Among Different Conduction Blocks

Block Type	CK-MB (ng/mL)	cTnI (ng/mL)	RBS (mg/dL)	TC (mg/dL)	TG (mg/dL)
First-degree heart block	21 ± 18.9	11.8 ± 13.2	143 ± 68.6	164 ± 49.8	120 ± 37.9
Left bundle branch block	20 ± 14.1	8.79 ± 5.56	140 ± 67.4	207 ± 51.1	133 ± 44.2
Mobitz type 1 heart block	20.3 ± 18.7	10.2 ± 9.80	138 ± 54.8	200 ± 44.9	129 ± 45
Mobitz type 2 heart block	15.7 ± 9.69	6.09 ± 5.67	146 ± 63.9	197 ± 44.2	148 ± 53.7
Right bundle branch block	72.5 ± 170	7.44 ± 5.67	139 ± 69.2	183 ± 62.5	166 ± 13.4
P*	.861	.447	.999	.157	.166

*Kruskal–Wallis test; cTnI, cardiac troponin I; CK-MB, creatine kinase; RBS, random blood sugar; TC, total cholesterol; TG, triglycerides.
